# An Overview of l-Amino Acid Oxidase Functions from Bacteria to Mammals: Focus on the Immunoregulatory Phenylalanine Oxidase IL4I1

**DOI:** 10.3390/molecules22122151

**Published:** 2017-12-05

**Authors:** Flavia Castellano, Valérie Molinier-Frenkel

**Affiliations:** 1 The Mondor Institute of Biomedical Research (IMRB), INSERM U955, Team 09, F-94010 Créteil CEDEX, France; 2 Faculty of Medicine, Paris Est University, F-94010 Créteil CEDEX, France; 3 Biological Resources Platform, Henri Mondor Hospital, AP-HP, F-94010 Créteil, France; 4 Department of Hematology-Immunology, Henri Mondor Hospital, AP-HP, F-94010 Créteil, France

**Keywords:** IL4I1, immunosuppression, l-amino acid oxidase

## Abstract

l-amino acid oxidases are flavin adenine dinucleotide-dependent enzymes present in all major kingdom of life, from bacteria to mammals. They participate in defense mechanisms by limiting the growth of most bacteria and parasites. A few mammalian LAAOs have been described, of which the enzyme “interleukin-4 induced gene 1” (IL4I1) is the best characterized. IL4I1 mainly oxidizes l-phenylalanine. It is a secreted enzyme physiologically produced by antigen presenting cells of the myeloid and B cell lineages and T helper type (Th) 17 cells. Important roles of IL4I1 in the fine control of the adaptive immune response in mice and humans have emerged during the last few years. Indeed, IL4I1 inhibits T cell proliferation and cytokine production and facilitates naïve CD4^+^ T-cell differentiation into regulatory T cells in vitro by limiting the capacity of T lymphocytes to respond to clonal receptor stimulation. It may also play a role in controlling the germinal center reaction for antibody production and limiting Th1 and Th17 responses. IL4I1 is expressed in tumor-associated macrophages of most human cancers and in some tumor cell types. Such expression, associated with its capacity to facilitate tumor growth by inhibiting the anti-tumor T-cell response, makes IL4I1 a new potential druggable target in the field of immunomodulation in cancer.

## 1. Introduction

The first evidence that natural amino acids are oxidized was presented in 1910 in a perfusion model [1]. The enzymatic stereospecific deamination of l-amino acids was subsequently observed in rat kidney and liver [1]. l-amino acid oxidases (LAAOs) are present in small or undetectable quantities in tissues of other mammals, suggesting that they do not play an important role in amino acid metabolism in this class. Similar enzymes have since been described in a wide variety of organisms, from bacteria to vertebrates, with LAAO from snake venoms receiving particular attention [2]. Vertebrate, fungal, and gastropod LAAOs belong to distinct phylogenetic groups, whereas bacterial LAAOs do not constitute a discrete group, but present phylogenetic relationships to LAAOs of other species and d-amino acid oxidases (DAAOs) [3]. In contrast to snake venom LAAOs, mammalian LAAOs remain poorly explored. In the last ten years, some of the functions of the mammalian phenylalanine oxidase IL4I1 have been deciphered and point to its role in the regulation of the adaptive immune response.

## 2. LAAO Structure and Reaction

**Figure 1:**
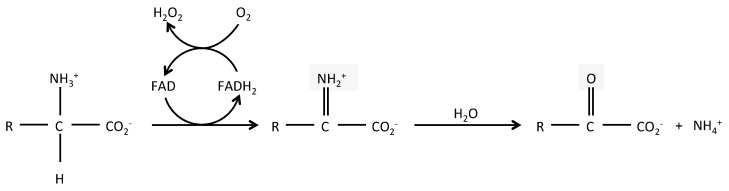
**Reaction catabolized by l-aminoacid oxidases**. Schematic representation of the reaction catabolized by LAAOs. R = amino acid specific group.

LAAOs (BRENDA EC 1.4.3.2) are mostly flavin adenine dinucleotide (FAD)-dependent enzymes and in some cases, dependent from the related cofactor flavin mononucleotide (FMN). FAD binding is shared with DAAOs, which are surprisingly, more widely expressed than LAAOs, despite catabolizing the uncommon D form of amino acids. The enzymatic reaction of LAAOs leads to the production of an α-keto acid, along with ammonia (NH_3_) and hydrogen peroxide (H_2_O_2_), from an l-amino acid (Figure 1). The crystal structure of several snake venom LAAOs has been resolved, allowing a better understanding of their catalytic mechanisms [4,5,6,7,8,9]. The reaction includes two steps: a first reductive reaction where an imino intermediate is formed through the transfer of a proton from the amino group of the substrate to the isoalloxazine ring of the FAD; the imino acid is next non-enzymatically hydrolyzed in its α-keto acid and ammonia. In a second step, the cycle is completed when the molecular oxygen enters into the catalytic site to re-oxidize the reduced FAD, leading to the production of hydrogen peroxide. LAAOs present a clear preference for hydrophobic amino acids, such as phenylalanine, tryptophan, tyrosine, and leucine [2], although they can catabolize most natural l-amino acids. Conversely, they are mostly strictly enantiospecific [4,9].

Studies on snake LAAOs have shown these enzymes to be active over a wide range of pH values, sometimes depending on the specific amino acid used as substrate [2]. They are stable at temperatures from 0 to 50 °C, but progressive inactivation occurs upon freezing to sub-zero temperatures (with the strongest inactivation between −15 and −30 °C), with the rate of inactivation depending on pH and buffer composition. Reversion of such inactivation is possible and has been shown to require a combination of lower pH and higher temperature [10].

The molecular weight of LAAOs is generally between 50,000 and 70,000 Da. The apparent molecular weight of the polypeptide is variably modified by *N*-glycosylations. The topology of LAAOs resembles that of polyamine oxidases, even if the identity between those enzymes stands at around 20%. It typically comprises three domains: a FAD-binding domain, a substrate-binding domain, and a helical domain. Each protomer is associated with a FAD molecule [7] and the FAD binding domain is composed of three discrete peptide sequences in the primary structure (residues 35–64, 242–318 and 446–486 in the *Calloselasma rhodostoma* protein). The color change of the crystalized protein from yellow to colorless indicates the reduced state of the LAAO-bound FAD. In *C. rhodostoma*, residues 5–25, 73–129, 233–236, and 323–420 make up the substrate-binding domain (phenylalanine). A second crystal structure of the LAAO from *C. rhodostoma* in association with phenylalanine shows the carboxylate group of the substrate bound to the cofactor through a salt bridge interaction with the guanidinium group of Arg90 and a hydrogen bond with the hydroxyl group of Tyr372 [6]. In the same crystallographic study, alternate conformations of the two active site residues H223 and R322, located at the entry of the channel for the substrate, have been suggested to be important for binding and release of substrate and product, respectively. Finally, the helical domain constitutes one side of a funnel-shaped entrance to the active site [7].

Biochemical studies have shown that LAAOs are present as dimers [2]. In the crystallized *Bothrops atrox* venom LAAO, the dimers are assembled asymmetrically with interactions between FAD- and substrate-binding domains of counterpart monomers. A conserved zinc-binding site at the interface between monomers may stabilize the dimer [5]. The crystal structure in a citrate or α-aminobenzoate complex of the Malayan pit viper enzyme has shown that four molecules are arranged in an asymmetric unit, as dimers of dimers [7]. However, more recent data obtained from *Bothrops atrox* venom LAAO suggest that this tetrameric structure is a consequence of the crystallization packing [5]. In contrast to snake venom LAAOs, in the bacterial *Rhodococcus opacus* LAAO, dimerization is due to a unique helical domain, whereas this enzyme lacks the glycosylation sites found in the same region in snake LAAOs [4]. No crystallography data are available for mammalian LAAOs. A modeling study performed using I-Tasser and Pymol programs suggests a 3-D globular structure of human IL4I1. The comparison with the *C. rhodostoma* protein indicates a similar composition in α and β helixes for most of the protein, with the exception of the C-terminal end of IL4I1, which should have a α-helix conformation, being absent in snake venom enzymes [11]. It should be noted that this C-terminal end is also highly divergent between IL4I1 from different mammalian species.

Snake venom LAAOs are heavily glycosylated and each protomer may contain up to 3.7 kDa of sugars [7,12]. Two glycosylation sites (Asn172 and Asn361) have been identified in *C. rhodostoma* and appear to be conserved in most other LAAOs [7,13]. MALDI-TOF analysis of the glycan moiety of *C. rhodostoma* shows a bi-sialylated, biantennary, core-fucosylated dodecasaccharide [12]. The glycosylation of LAAOs is important for the solubility, secretion and in some cases activity of these enzymes. Indeed, treatment with tunicamycin, which blocks *N*-linked glycosylation, of Apoxin I, an LAAO from western diamondback rattlesnake venom, and the mammalian LAAO, Interleukin-4 induced gene 1 (IL4I1), reduces the activity and secretion of these proteins [14,15,16]. Moreover, these sugar moieties have been suggested to participate indirectly in the toxicity of some LAAOs [17].

The enzymatic activity of LAAOs can be blocked by specific inhibitors, amongst which benzoic acid and its derivatives have *Kis* values in the millimolar range [18]. Apart from being relatively inefficient, benzoic acid does not appear to be very specific, as it can block the activity of peroxidase (Castellano et al. data not shown), a relay enzyme often used in LAAO activity measurements. Other inhibitors have been described, such as aristolochic acid [19] and tryptophan derivatives [20]. Finally, some molecules, such as l-propargylglycine, can irreversibly inhibit snake venom LAAOs [21].

## 3. LAAO Expression and Functions

As already stated, LAAOs are widely expressed both in prokaryotes and eukaryotes and share high similarity in the active site. Several bacterial species (including *Escherichia coli*, *Bacillus subtilis*, *Rhodococcus opacus*, and *Proteus mirabilis*) produce LAAOs that may have specificity for multiple aliphatic and aromatic l-amino acids [22] or, in certain cases, can metabolize only a relatively narrow range of basic l-amino acids. It has been suggested that the enzyme produced by prokaryotes has antibacterial activity and participates in interspecies competition [23]. Similar anti-microbial activity has been reported for LAAOs present in mammalian cells (see below). LAAO expression in filamentous fungi, such as *Aspergillus nidulans*, instead enables these organisms to scavenge nitrogen from amino acids present in soil for their metabolic functions [24,25]. LAAO activity in higher fungi is concentrated in fruiting bodies and reported to be present in highly toxic species of *Amanita phalloides* and *Clitocybe geotropa* [26,27].

LAAOs have also been described in animals. For example, they are used as a defense mechanism in mollusks and some fish [28,29,30]. It has been suggested that Escapin, an LAAO produced by *Aplysia californica*, might have both antibacterial and anti-predatory functions [31]. Chub mackerel LAAO, apoptosis inducing protein (AIP), is produced only when larvae of the parasite Anisakis simplex infect the fish. AIP is localized in capsules formed around the larvae and believed to be necessary to contain the infection [29].

In vertebrates, reptile LAAOs have been extensively studied. They represent a major component of snake venoms from Viperidae and Elapidae, including members of the genera *Crotalus*, *Bothrops*, *Vipera*, *Calloselasma*, and *Bungarus,* and may participate in their toxic effects. The large availability of snake venom has allowed multiple studies. Snake venom LAAOs have been shown to mediate apoptosis of several mammalian cell types by activating caspases and inducing the expression of proapoptotic proteins [32,33]. High levels of hydrogen peroxide produced by the activity of LAAO is responsible for the induction of necrosis, whereas apoptosis may be mediated by internalization of the enzyme after binding via its glycan moiety [17] and may be independent of its enzymatic activity [34]. Antiproliferative and/or cytotoxic effects on tumor cell lines have led investigators to propose their use as antitumor therapeutics [35,36]. Such applications should take into account the natural antigenicity of these proteins and their proinflammatory properties [37]. Snake venom LAAO also activates or inhibits platelet aggregation, depending likely on the experimental procedures [38,39], and can provoke hemorrhage. Apart from their toxicity towards endothelial cells, coagulation defects might result from inhibition of the activity of coagulation factor IX [40], but this latter effect is still a matter of debate [41]. Finally, snake venom LAAO can cause hemolysis and edema [42] and displays anti-bacterial activity, like all LAAOs described so far [43].

Most of the reported functions of LAAO have been attributed to oxidative stress due to H_2_O_2_ production. In particular, H_2_O_2_ seems responsible for cellular apoptosis, platelet aggregation, and some of the antibacterial and anti-parasitic properties, since the use of glutathione or catalase can limit all these effects in vitro [38,44,45,46]. In a study of the mammalian LAAO IL4I1, basification of the extracellular medium by NH_3_ (another byproduct of the reaction) has been shown to amplify the H_2_O_2_-induced bactericidy (See “IL4I1, an LAAO implicated in immune regulation”). NH_3_ might also be involved in the potentiation of other H_2_O_2_-mediated biological effects. There is currently no known biological effect of the α-keto acid produced by the enzymatic reaction.

## 4. Mammalian LAAOs

Although initially described in mammalian liver and kidney, a few reports have described LAAOs in other tissues in this class of animals. LAAOs have been found in measurable quantities in four compartments in mammals: spermatozoa of various species [47,48,49], brain [50,51], milk [52], and the immune system [53]. The sperm enzyme has a preference for aromatic amino acids and arginine. This LAAO is found in the head of the spermatozoa and is released in preparations with high sperm mortality, where it has been shown to limit sperm motility [47]. Two types of LAAO have been described in brain: a lysine oxidase, which is involved in the pipecolic acid pathway [50], and isoform 2 of Interleukin 4-induced gene 1 (IL4I1) [51] (see IL4I1 an LAAO implicated in immune regulation). Milk LAAO has a large spectrum of amino acid substrates and its mRNA is strongly induced at the late stage of pregnancy and during lactation in mice [52]. Mice deficient for the production of milk LAAO in the mammary gland have a lower capacity to control infection than WT mice when injected with *Staphylococcus aureus*, showing both more severe clinical manifestations and increased mortality. This shows an anti-bacterial function of the milk enzyme, which might be of varying importance, depending on the species, as the level of LAAO in cow’s milk is lower than that of mice. Nevertheless, the increase of cow LAAO mRNA during mastitis suggests that the anti-bacterial function is preserved [54]. Apart from their anti-infectious functions, mammalian LAAOs may have evolved to become a component of the immune system where they regulate specific immune functions, as exemplified by IL4I1 [55].

## 5. IL4I1, an LAAO Implicated in Immune Regulation

IL4I1 has received this peculiar name because of the induction of its mRNA in mouse B splenocytes being stimulated by the cytokine interleukin 4 (IL-4) [53]. The high identity of the *IL4I1* gene to known LAAOs (43% with AIP and 37% with Apoxin I) led investigators to suspect the IL4I1 protein of sharing this function, which was later confirmed in mice and humans [56,57]. Since then, five isoforms of *IL4I1* have been reported in DNA databases, all leading to production of a similar protein; the only differences being in the 5’ untranslated region and the first two exons that code for a signal peptide. Isoform 1 is restricted to lymphoid tissues [56]. The second isoform is highly expressed in Sertoli cells at the periphery of the testis ducts and in rare cells of the nervous system (such as Purkinje cells in the cerebellum, mitral cells of the olfactory bulb, and cells of the hippocampus) [51]. 

**Figure 2:**
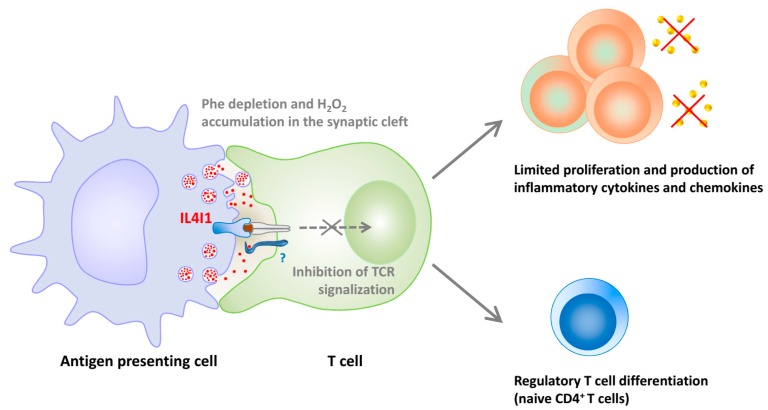
**Regulation of T cell proliferation, functions, and differentiation by IL4I1.** IL4I1 is released in the synaptic cleft from granules accumulated in mature antigen presenting cells. The enzyme binds to the surface of T cells and blocks signaling downstream of the T cell receptor (TCR). The diminished T cell activation leads to impaired proliferation and capacity to produce Th1 cytokines and proinflammatory chemokines. Moreover, naïve CD4^+^ T cells exposed to IL4I1 preferentially differentiate into regulatory T cells if not stimulated by strong cytokine signals. Inhibition of effector T cell activation and increased differentiation of regulatory T cells result in a state of immunosuppression.

Our group has shown that mouse and human IL4I1 are glycosylated and secreted LAAOs that preferentially degrade phenylalanine and, to a lesser extent, arginine [11,16]. IL4I1 limits T lymphocyte activation and proliferation [16,58], in part via the production of H_2_O_2_ (Figure 2). A recent interaction of IL4I1 with T lymphocytes has been shown, which could participate in its inhibitory functions, either by concentrating the enzyme at the T cell surface or by mediating a negative intracellular signal [58].

**Figure 3:**
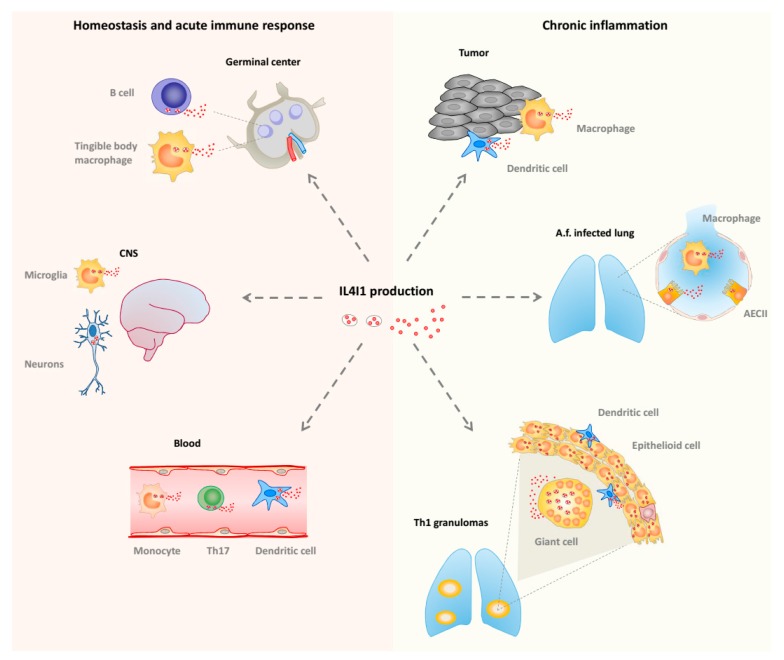
**IL4I1 production in vivo**. **Left**: homeostatic conditions and acute immune response. IL4I1 is detected in secondary lymphoid organs, particularly in germinal center B cells and tingible body macrophages during the development of the T-cell-dependent antibody response. The isoform 2 of IL4I1 is present in rare cells of the central nervous system (CNS). In the blood, IL4I1 is naturally produced by monocytes, dendritic cells, and T helper type 17 (Th17) cells. **Right**: chronic inflammatory conditions. IL4I1 is detected in Th1 inflammatory lesions including sarcoidosis and tuberculosis granuloma. A high density of IL4I1 granules is present in dendritic cells and macrophage-derived epithelioid and giant multinucleated cells. In the lung of *Aspergillus fumigatus* (Af)-infected individuals, IL4I1 is also produced by type II alveolar epithelial cells (AECII). In tumors, IL4I1 is secreted by infiltrating dendritic cells and macrophages, regardless of the tumor type. IL4I1 is also expressed by some tumor cell types, such as germinal center derived B-cell lymphoma (not depicted).

In humans, IL4I1 is expressed in monocytes/macrophages and dendritic cells present in chronic T helper type 1 (Th1) inflammation, such as sarcoidosis or tuberculosis granulomas, and tumors (Figure 3). It has also been reported in alveolar type II cells during infection by the mold *Aspergillus fumigatus* [59]. In accordance with these observations, the induction of IL4I1 expression is mediated by inflammatory and Th1 stimuli, such as pathogen-associated molecular patterns (ligands for Toll like receptors), tumor necrosis factor (TNF) α, and interferons (IFNs), acting via NFκB and STAT1. IL4I1 may limit local Th1 inflammation or participate in its resolution by decreasing the production of inflammatory chemokines, IL-2, and IFNγ [60,61] or by limiting the growth of the pathogen, as IL4I1 also presents some ancestral anti-bacterial function [62]. Intriguingly, IL4I1 expression in mouse macrophages was reported to be controlled by Th2 type stimuli [63].

IL4I1 is also expressed by peripheral blood B cells stimulated by IL-4 and CD40L, which activate the STAT6 and/or NF-κB pathways, respectively [60], and has been detected in germinal center B cells, i.e., B cells involved in a T-cell-dependent immune response [64,65] (Figure 3). In accordance, we recently demonstrated a key role of IL4I1 in modulating B cell differentiation, particularly at the germinal center stage [66]. IL4I1 has also been detected in B cell lymphomas originating from the germinal center [67,68], mirroring this natural expression. Furthermore, as stated above, IL4I1 is strongly expressed by tumor-associated macrophages of most types of cancer and has been proposed to play a role in the escape of tumors from specific immune responses. Indeed, expression of IL4I1 in transplanted tumors or in a mouse model of spontaneous melanoma correlates with a diminished anti-tumor immune response, reduction of the T cell infiltrate, and enhanced aggressiveness of the tumor [69,70].

IL4I1 has also been detected in some CD4^+^ T cell types. In particular, it is expressed by Th17 cells or T cells undergoing Th17 differentiation, under the control of the RORγT master gene, in which it limits cell-cycle progression and thus pathogenicity of this highly proinflammatory cell type [71,72,73] (Figure 3). Moreover, IL4I1 biases naive CD4^+^ T cell differentiation towards that of FoxP3^+^ regulatory T cells [74]. Thus, IL4I1 may regulate the balance of effector versus suppressive T cells in inflammatory microenvironments, such as cancer [60,74]. In accordance with this function, the *IL4I1* gene was shown to be associated with a poor prognosis in a transcriptomic study of the micro-dissected tumor stroma of human breast cancers [75].

## 6. Conclusions

LAAO represents a family of enzymes expressed in all major kingdoms of life from prokaryotes to vertebrates. Their biological functions remain only partially characterized. One established function is their role in anti-infectious defense. Direct toxic effects on microorganisms may have evolved to become regulatory functions of the adaptive immune response in higher vertebrates, including humans. IL4I1 is the best characterized mammalian LAAO and displays such immunoregulatory functions. Manipulating IL4I1 in vivo opens new avenues in the control of pathological inflammatory responses. For example, the chemical inhibition of IL4I1 activity may represent a new adjuvant strategy for the treatment of cancer by restoring specific anti-tumor immune responses.

## Abbreviations

LAAOs	l-amino acid oxidases
DAAOs	d-amino acid oxidases
FAD	Flavin Adenine Dinucleotide
FMN	Flavin Mononucleotide
FoxP3	Forkhead box P3
IFN	Interferon
IL	Interleukin
IL4I1	Interleukin-4 Induced gene 1
NFκB	Nuclear factor-kappa B
RORγT	Retinoic acid receptor-related orphan receptor gamma
STAT	Signal Transducers and Activators of Transcription
Th	T helper
TNF	Tumor necrosis factor
